# Supplementary material for: Antioxidant Enzyme-Mimetic Activity and Neuroprotective Effects of Cerium Oxide Nanoparticles Stabilized with Various Ratios of Citric Acid and EDTA
Source: Biomolecules. 2019 Oct 3;9(10):562. doi: 10.3390/biom9100562 (PMC6843313; doi:10.3390/biom9100562)
Supplement: Supplementary file 1 [file biomolecules-09-00562-s001.pdf]

Supplementary Materials:

Antioxidant enzyme-mimetic activity and neuroprotective effects of cerium oxide nanoparticles stabilized with various ratios of citric acid and EDTA

[#1,2]Ana Y. Estevez, [#3,4]Mallikarjunarao Ganesana, [5]John F. Trentini, [5,6]James E. Olsen, [5]Guangze Li, [1]Yvonne O. Boateng, [1]Jennifer Lipps, [1]Sarah E.R. Yablonski, [3]William T. Donnelly, [3]James C. Leiter, and [1]Joseph S. Erlichman

[#]Contributed equally

Affiliations:

[1]Biology and [2]Psychology Departments, St. Lawrence University, Canton NY 13617

[3]Department of Molecular and Systems Biology, Geisel School of Medicine at Dartmouth, Hanover, NH 03755.

[4]Current Address: Department of Chemistry, University of Virginia, Charlottesville, VA, USA.

[5]Department of Emergency Medicine, Wright State University, Boonshoft School of Medicine, Dayton, OH 45435

[6]Department of Neuroscience, Cell Biology, and Physiology, Wright State University, Boonshoft School of Medicine, Dayton, OH 45435

Corresponding author: Ana Y. Estevez, St. Lawrence University, 23 Romoda Drive, Canton NY 13617 aestevez@stlawu.edu

Supplementary Methods

Analytical Performance of Superoxide Microbiosensor *in vitro*

The performance characteristics of cytochrome C (Cyt C) modified, gold wire electrodes, such as reactivity, sensitivity and selectivity towards superoxide radical (SO), were evaluated by the measurements of SO generated in a hypoxanthine/xanthine oxidase (HX/XOD) system in aCSF (124 mM NaCl, 3 mM KCl, 2.4 mM $CaCl_2$, 1.3 mM $MgSO_4$, 1.24 mM KH_2PO_4, 26 mM $NaHCO_3$, 10 mM glucose, pH 7.4, 300 mOsm). Microbiosensor performance was tested at a concentration of 100 μM hypoxanthine, and superoxide was generated enzymatically by oxidizing HX. XOD catalyzes the oxidation of HX in the presence of molecular oxygen to uric acid and H_2O_2, and the superoxide radical anion is formed as an intermediate product [1]. The superoxide generated can be detected based on the reversible electrochemistry of Cyt C, a single electron transport protein derived from mitochondria, immobilized onto the modified electrode. The presence of superoxide can be detected amperometrically at an applied potential of +0.15 V at the Cyt C-modified sensor surface. Under these conditions, a steady-state sensor signal can be obtained that reflects the rate of generation and dismutation of superoxide. The current response of the sensor is proportional to the square root of XOD activity [2]. The increase in current response of the sensor after the addition of XOD is indicative of the superoxide generated. A dose-dependent increase in superoxide concentrations can be demonstrated by adding varying amounts of XOD. A linear increase in sensor signal after XOD additions demonstrates the dependence of the sensor signal on XOD activity.

Figure S2A shows an overlay of multiple amperometric responses of the Cyt C biosensor generated following the addition of variable amounts of XOD to the reaction cell containing HX. The stable current signal before the addition of XOD was defined as the baseline superoxide radical current. The signal response of the biosensor to the superoxide radical was calculated as

the change in current following the addition of XOD to the point it reached a plateau. A current response was seen in less than 1 sec after XOD injection, and a steady-state limiting current was reached within 4-5 sec.

Figure S2B shows the dependence of the sensor signal (expressed as experimental current measured or the equivalent calculated superoxide concentration) on XOD activity. The amperometric sensor signal was proportional to the square root of the XOD up to 80 mU/mL enzyme activity, which was equal to a superoxide concentration of 1.23 μM. The linear range of biosensor for superoxide was 0.00 - 1.23 μM with a sensitivity of 11.65 nA/ μM superoxide. The detection limit of the microbiosensor was 10.5 nM, which was calculated according to the 3 sigmas/R′ criteria (R′ is the slope of the linear calibration curve and sigma is the standard deviation of the amperometric signal of the blank solution). Overall the sensor performed well even after a 10-fold miniaturization (reduced from 1.5 cm to 1.5 mm) compared with our previous sensors [3].

The selectivity of the biosensor toward the superoxide radical was confirmed by the addition of a natural antioxidant SOD to inactivate the superoxide radical. After enzymatic generation of superoxide at a concentration of 0.93 μM (the calculated concentration), addition of 10 U/mL of SOD completely suppressed the electrochemical signal (Figure S3). Thus, the sensor demonstrated good specificity towards superoxide and no interference from the electrochemically active by-products of the reaction. The biosensor also exhibited good functionality and was stable after up to 7 days storage with no significant change in responsiveness when stored in aCSF at +4 °C.

The microbiosensors used for *in vivo* measurements were calibrated in aCSF using a six-point calibration *in vitro* before and after implantation. Figure S4 shows corresponding calibration curves for the Cyt C biosensors before and after implantation. The biosensors lost, on average, 65.7 ± 11.5% (n=13) of their activity after implantation. Pre-calibration measurements were used to estimate the superoxide concentration for the amperometric signal responses observed *in vivo.*

References

(1) Fujita, M.; Tsuruta, R.; Kasaoka, S.; Fujimoto, K.; Tanaka, R.; Oda, Y.; Nanba, M.; Igarashi, M.; Yuasa, M.; Yoshikawa, T.; Maekawa, T. In vivo real-time measurement of superoxide anion radical with a novel electrochemical sensor. *Free Radic. Biol. Med.* **2009***, 47*, 1039-1048.

(2) McCord, J. M.; Fridovich, I. The Utility of Superoxide Dismutase in Studying Free Radical Reactions: I. Radicals generated by the interaction of sulfite, dimethyl sulfoxide, and oxygen. *J. Biol. Chem.* **1969***, 244*, 6056-6063.

(3) Ganesana, M.; Erlichman, J. S.; Andreescu, S. Real-time monitoring of superoxide accumulation and antioxidant activity in a brain slice model using an electrochemical cytochrome c biosensor. *Free Radic. Biol. Med.* **2012***, 53*, 2240-2249.

Supplementary Figures

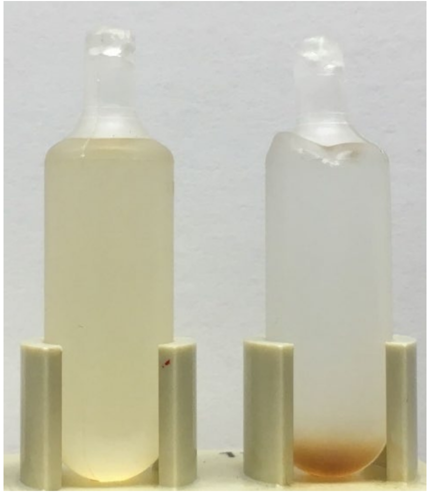

Figure S1. Photograph of one of the nanoparticle formulations before (left) and after (right) centrifugation at 4.3 X 10^5 g for 1 h.

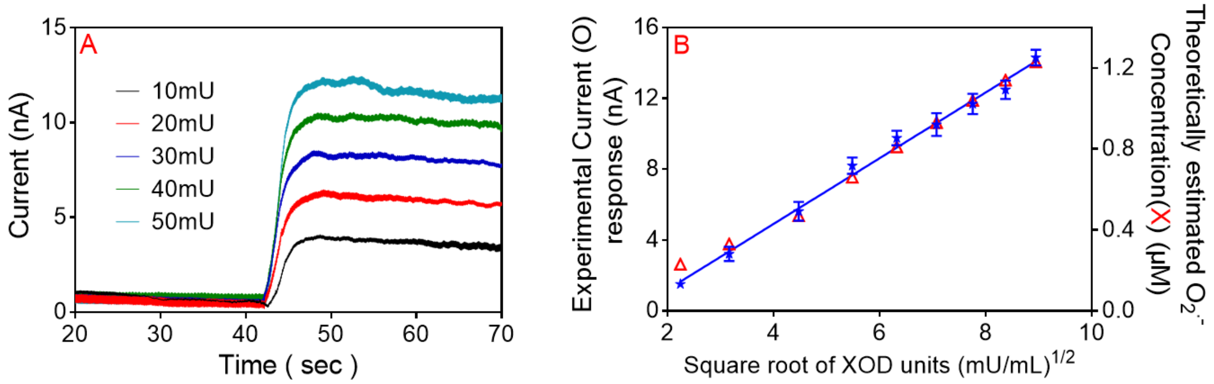

Figure S2. Performance and calibration of the microbiosensor. (A) Performance of Cyt C microbiosensor for the detection of superoxide anion radical was tested in vitro in aCSF. Superoxide anion radical current generated by the addition of XOD concentrations 10, 20, 30, 40 and 50 mU in the presence of 100 µM HX, at an applied potential of +0.15 V. Superoxide current response was estimated as the difference between the baseline-defined as the stable state before the addition of XOD and plateau-current response reached maximum after the addition of XOD. (B) Calibration and linear range of the biosensor showing the dependence of the experimental sensor signal (O) and the theoretically estimated steady-state superoxide concentration (x) on XOD activity in aCSF. The HX concentration was 100 µM and XOD concentrations were 5, 10, 20, 30, 40, 50, 60, 70 and 80 mU/mL.

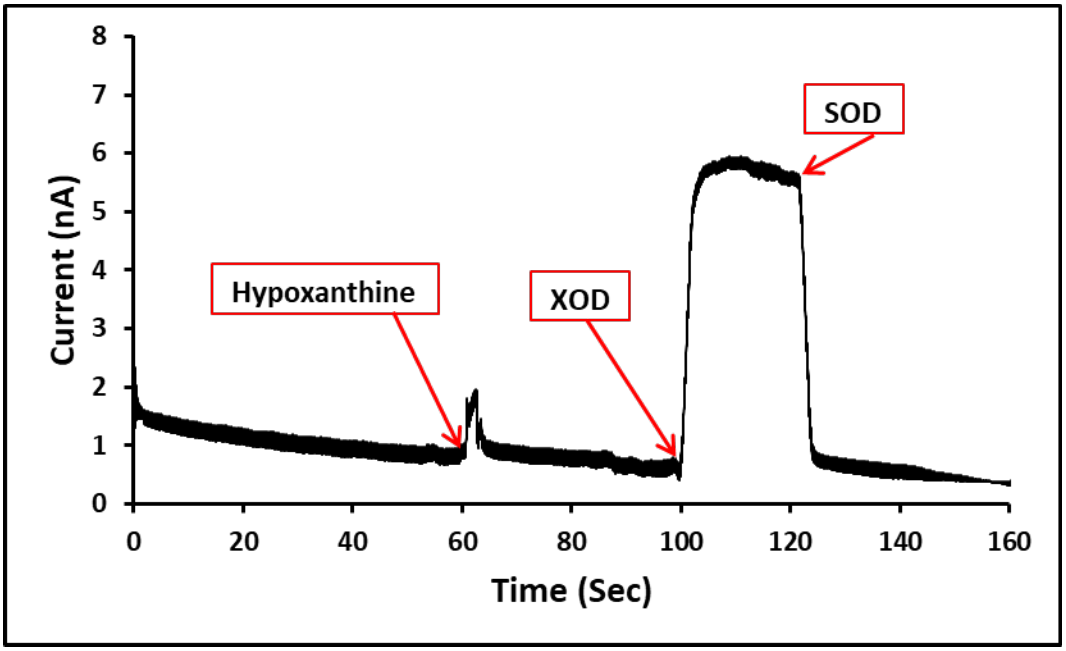

Figure S3: Superoxide anion radical current generated by the addition of 100 μM HX and 20 U/ml XOD in aCSF at an applied potential of +0.15 V. Current response was diminished by the addition of natural antioxidant to superoxide, 10 U/ml SOD, which demonstrates the specificity of the signal to superoxide.

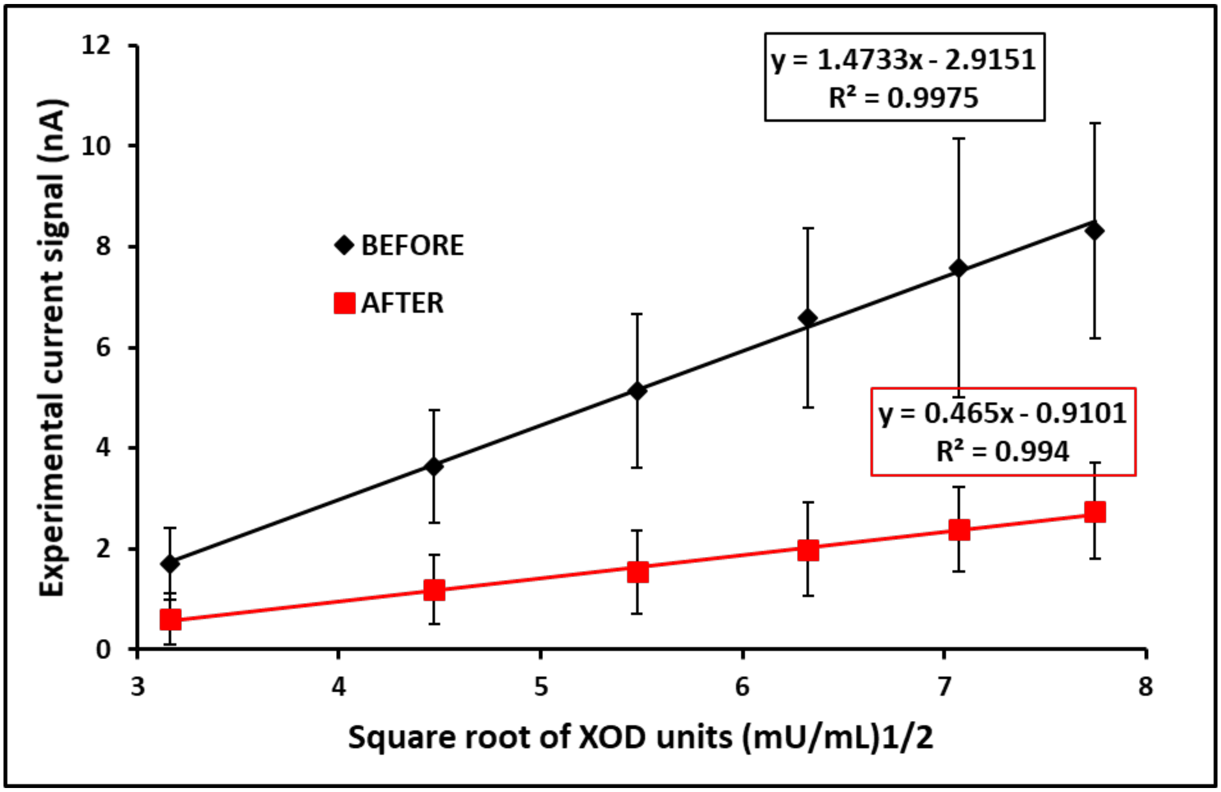

Figure S4. Biosensor sensitivity in aCSF. Six-point calibration showing the sensitivity of biosensors before and after *in vivo* testing. The biosensors lost an average of 65.7 ± 11.5% (n=13) of their sensitivity once they used for *in vivo* testing. Sensors calibration was performed with a constant HX concentration of 100 µM and XOD concentrations of 10, 20, 30, 40, 50 and 60, mU/mL.
